# Noise signature on interval timing

**DOI:** 10.1186/1471-2202-16-S1-P180

**Published:** 2015-12-18

**Authors:** Sorinel A Oprisan, Derek N Novo

**Affiliations:** 1 Department of Physics and Astronomy, College of Charleston, Charleston, SC 29424, USA

## 

Among other essential adaptations is the capability of organisms to estimate durations in the seconds-to-hours range (interval timing). Such capabilities are critical for fundamental cognitive processes like decision making, rate calculation, and planning of action [1]. In their seminal work on computational modeling of interval timing, Matell and Meck [2] revitalized the striatal beat frequency (SBF) model that utilizes the coincident activation of a series of oscillators to code for different durations. They showed through numerical simulations that the SBF model is capable of reproducing two of the interval timing signatures: (a) precise timing, i.e. the model output peaks at the training/criterion time and (b) scalar timing, i.e. the error in timing increases linearly with the criterion time. The SBF model was capable of producing scalar timing only when different types of biologically realistic variances (frequency, memory, etc.) were considered [3].

**Figure 1:**
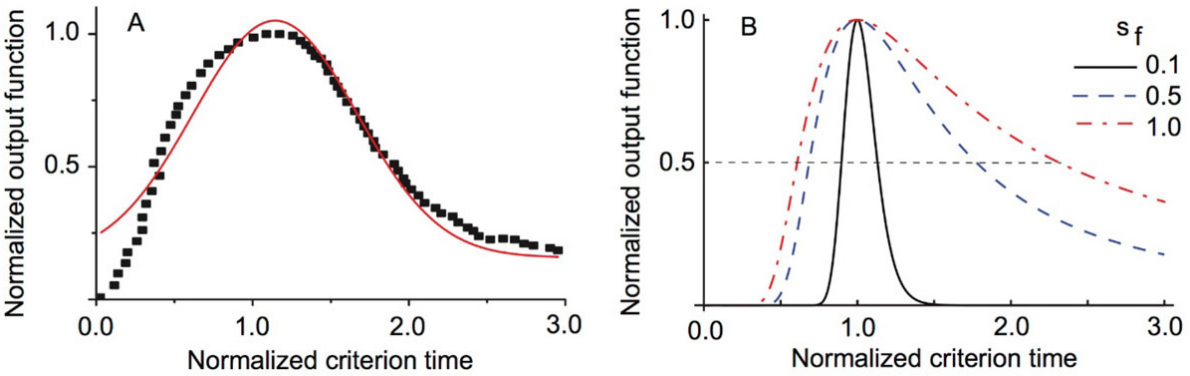
**A. Normalized response for mice (squares - redrawn after **[4]) **versus normalized criterion time and the corresponding best Gaussian fit (continuous line)**. **B**. Theoretical output from SBF model with different frequency variances.

In this work, we investigated what effect each type of variance/noise has on the shape of the SBF model output. In particular, we noticed that the experimentally measured response rate is not quite Gaussian (see Figure 1A) and instead has a long tail. Mathematically, the output function of set of coincidental (sinusoidal) oscillators is given by [3]:

$$out\left(t\right)=\sum cos\left(2\pi f_{i}T\right)\;cos\left(2\pi f_{i}t\right),$$

where *T *is the criterion time, *f_i _*are the frequencies of neural oscillators. The criterion time *T *is learned during the training phase and stored/retrieved from the long-term memory with some errors. The firing frequencies *f_i _*of all neural oscillators also fluctuate. We found that memory variance (*s_T_*) preserves the Gaussian shape of the output function, whereas the frequency variance (*s_f_*) skews and has a long tail similar to experimental observations (see Figure 1B).

In addition to the significantly difference contributions to the shape of output functions, we also found that memory and frequency noises shift the peak of the Gaussian. Memory noise affects the storage/retrieval of criterion *T *and shifts the peak of the output to the right, i.e. *t_T _= T(1+_T_)*, where _T _is a number that depends on the range of stored values of *T *and the probability distribution function (*pdf_T_*) of the noise. Frequency variance shifts the peak of the output to the left, i.e. *t_f _= T/(1+_f_)*, where _f _is a number that depended on the range of frequencies and the *pdf_f _*of the noise.
